# Technical and Psychosocial Challenges of mHealth Usage for Antiretroviral Therapy Adherence Among People Living With HIV in a Resource-Limited Setting: Case Series

**DOI:** 10.2196/14649

**Published:** 2020-06-10

**Authors:** Kennedy Michael Ngowi, Furaha Lyamuya, Blandina T Mmbaga, Eva Muro, Zawadiel Hillu, Mary Shirima, Rob E Aarnoutse, Mirjam AG Sprangers, Pythia T Nieuwkerk, Peter Reiss, Marion Sumari-de Boer

**Affiliations:** 1 Kilimanjaro Clinical Research Institute Kilimanjaro Christian Medical Centre Moshi United Republic of Tanzania; 2 Department of Medical Psychology Amsterdam University Medical Centers Amsterdam Netherlands; 3 Kilimanjaro Christian Medical Centre Moshi United Republic of Tanzania; 4 Kilimanjaro Christian Medical University College Moshi United Republic of Tanzania; 5 Majengo Dispensary Moshi United Republic of Tanzania; 6 Radboud Institute for Health Sciences & Department of Pharmacy Radboud University Medical Center Nijmegen Netherlands; 7 Department of Global Health and Division of infectious Diseases Amsterdam institute of Global Health and Development Amsterdam University Medical Centers Amsterdam Netherlands; 8 Stichting HIV Monitoring Hogeschool van Amsterdam Amsterdam Netherlands

**Keywords:** mHealth, case series, adherence, HIV, real-time medication monitoring, SMS, antiretroviral therapy

## Abstract

**Background:**

Mobile communication has been found to improve antiretroviral therapy (ART) adherence among people living with HIV. In an ongoing randomized clinical trial, 2 mobile communication strategies (ie, sending SMS text messages and real-time medication monitoring [RTMM]) were used to improve adherence to ART among people living with HIV in Tanzania. We noticed remarkable discrepancies between self-reported adherence and adherence recorded by SMS text messaging or RTMM among some of the first trial participants.

**Objective:**

Our objective was to describe these cases and the observed discrepancies in more detail, to serve as a useful illustration of some of the challenges in using mobile health in resource-limited settings.

**Methods:**

In an ongoing randomized trial, adults living with HIV from two HIV treatment centers in Tanzania who were suspected of low levels of adherence were randomly assigned in a 1:1:1 ratio to receive (1) SMS text message reminders, (2) an RTMM device, or (3) no additional intervention to standard HIV care. During bimonthly study visits, the participants self-reported their level of adherence, received feedback about their level of adherence based on SMS text messaging or RTMM, and discussed strategies to overcome adherence problems with nurses providing HIV care. For the purpose of this report, we selected people living with HIV who had completed 5 follow-up visits and consistently reported more than 95% adherence, while SMS text messaging or RTMM recorded lower than 75% adherence. The participants were invited for a short, face-to-face in-depth interview to explore reasons for this discrepancy.

**Results:**

At the time of this analysis, 26 participants had completed follow-up. Six of these evidenced the above-mentioned discrepancies, with an average adherence of 46% based on SMS text messaging or RTMM, while self-reported adherence was 98%. Five of these 6 participants insisted that their adherence to ART was good, with 4 reporting that their adherence to properly using the monitoring device was low. Three participants mentioned concerns about involuntary disclosure of HIV status as a main reason for low adherence to using the device. Two participants were still depending on other reminder cues despite receiving SMS text message or RTMM reminders. Poor network coverage caused low adherence in 1 participant.

**Conclusions:**

Psychosocial barriers were reported as importantly contributing to low adherence, both with respect to use of ART and proper use of the adherence-monitoring device. This case series illustrates that when introducing new digital adherence monitoring technology, researchers should consider psychosocial barriers and distinguish between adherence to device use and adherence to treatment.

**Trial Registration:**

Pan African Clinical Trials Registry PACTR201712002844286; https://tinyurl.com/y98q4p3l

## Introduction

The Kilimanjaro region in Tanzania has an HIV prevalence rate of 2.6%, which is lower than the country’s national prevalence of 4.5% [1,2]. However, in this region, of those who receive antiretroviral treatment (ART), only 47% of people living with HIV have viral load suppression [1]. This suggests that adherence to ART is often not optimal. Due to limited resources, viral load is not routinely monitored, and consequently, timely information about potential poor adherence to ART is not available to HIV health care providers. Therefore, alternative means of providing information about and interventions to improve adherence to ART among people living with HIV are needed [3]. Mobile phone technology can potentially help to fill this need by using digital tools.

Several digital tools exist to monitor and intervene on adherence to treatment. Real-time medication monitoring (RTMM) records the date and time of every opening of a pillbox, a so-called event. It sends this information in real time to a web platform via the mobile network and is one of the existing technologies for monitoring adherence. Reminder SMS text messages may be sent to people living with HIV if the pillbox is not opened on time [4]. Text messaging can also be used on its own as another mobile phone technology to monitor and improve adherence. The feasibility of text messages was found to have mixed results, especially in rural areas [5,6]. Previous studies have described several challenges of delivering RTMM and SMS text messaging in resource-limited settings, such as poor network coverage, power failure, and lack of interoperability among network providers [5,7]. However, previous studies did not describe in detail how such challenges affect the delivery and use of mobile health (mHealth) strategies for treatment adherence. Furthermore, despite these technical challenges, we found that it was feasible and acceptable to monitor ART adherence using RTMM among people living with HIV who reside in the Kilimanjaro region [8].

Currently, we are conducting a randomized trial in which we investigate the effect of mHealth strategies to improve adherence to treatment among people living with HIV in the Kilimanjaro region. People living with HIV are randomly assigned in a 1:1:1 ratio to receive (1) SMS text message reminders, (2) an RTMM device, or (3) no additional intervention to standard HIV care. During the trial, we encountered major discrepancies between self-reported adherence and adherence reports generated by the assigned mHealth intervention in several of our trial participants. The objective of this report is to describe these cases and the observed discrepancies in more detail to serve as a useful illustration of some of the challenges researchers and health care providers need to consider when intending to use mHealth interventions to enhance adherence to ART in resource-limited settings.

## Methods

### Patients Enrolled in the Randomized Controlled Trial

We first describe the trial and the interventions from where we selected our patients. Our ongoing, parallel-group, 3-arm randomized controlled trial (REMIND Study) has enrolled adults living with HIV from the Kilimanjaro Christian Medical Centre and the Majengo Health Centre, 2 specialized HIV care and treatment centers in Moshi, Tanzania. By 2018, the Kilimanjaro Christian Medical Centre served a total of 2800 people living with HIV, while Majengo served 1200 people living with HIV. In our larger randomized controlled trial study, 264 people living with HIV were screened from both health centers. The study nurses played a vital role in ensuring that potential participants would understand the study. Written informed consent was requested from participants, who were subsequently randomly assigned equally to the study arms SMS text messaging, RTMM, and control. The study was approved by the Kilimanjaro Christian Medical College Research Ethics and Review Committee and the National Health Research Ethics Sub-Committee of Tanzania.

The main eligibility criteria for enrollment in the trial were that the participant must currently be receiving ART and that there is a suspicion of low adherence levels. The suspicion of low adherence was based on the following criteria, as subjectively judged by nurse counselors: (1) self-reported limited adherence, (2) missed medication refill visit, (3) return of leftover medication, or (4) other signs of nonadherence**,** including continuous high viral loads. Exclusion criteria were hospital admission or participation in other trials.

### Intervention Arms

#### SMS Text Messaging

**Figure 1:**
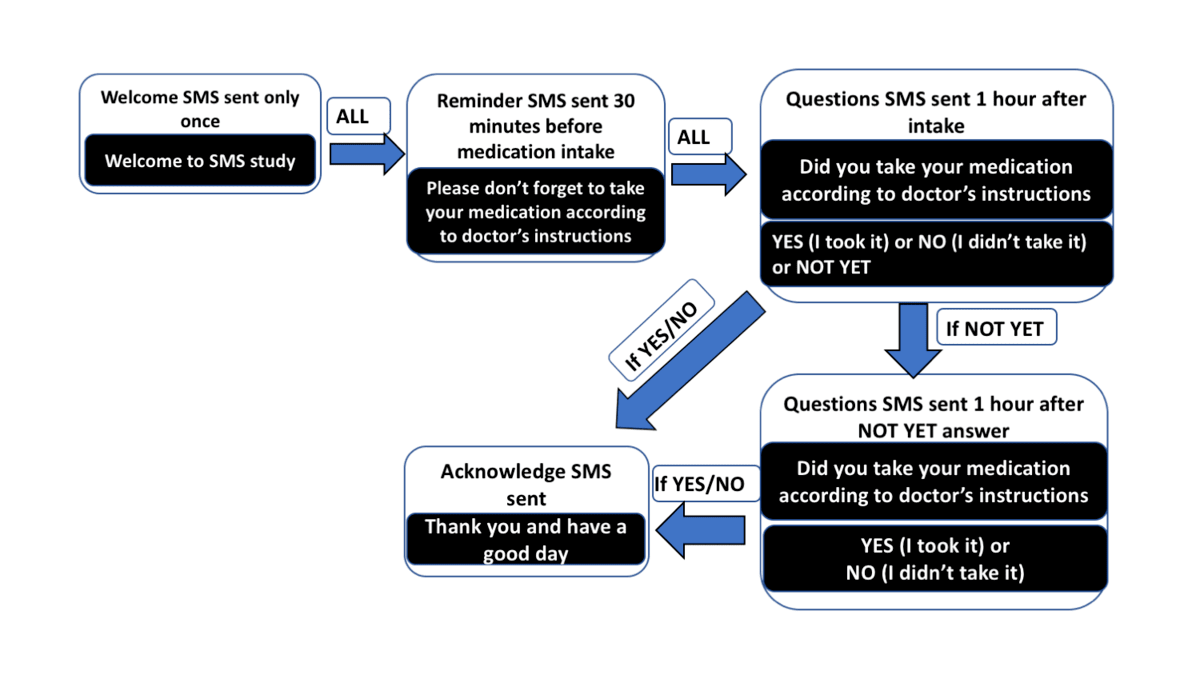
Flow of SMS text messages.

All participants who were enrolled in the SMS arm received 3 messages weekly on random days. The messages were in Swahili and were similar for all patients. Thirty minutes before the usual time of intake, an SMS text message was sent to remind patients to take their medication. One hour after the usual time of intake, another SMS text message was sent with the question “Did you take your medication?” The participant was asked to reply, choosing from the following 3 options: (1) Yes, I took it, (2) No, I did not take it, or (3) Not yet. The flow of SMS text messages is depicted in Figure 1.

#### RTMM

**Figure 2:**
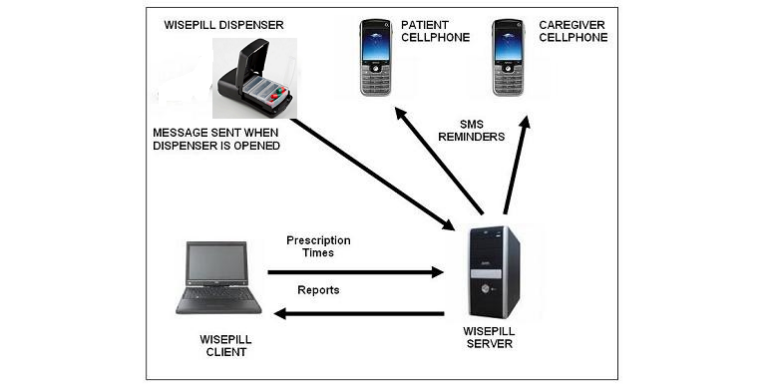
Wisepill device architecture. (Source: Wisepill Technologies).

All participants enrolled in the RTMM arm received a Wisepill device (Wisepill Technologies) containing a third-generation SIM card that sends signals to a central server each time the device is opened, a so-called medication event and proxy for medication intake. The signal contains information about the time the device is opened, identification number of the device, and technical information about the battery and strength of the signal. If the device was not opened within 1 hour and 15 minutes after the usual time of intake, the participant receives an SMS text message with a question about whether they took their medication. The respondent could not respond to the text message because the device does not have a function to support incoming SMS text messages from users. Authorized members of the study team could access the adherence report generated by the device by signing in through the protected web portal. Figure 2 presents the flow of communication between the pillbox, central server, and a patient’s phone.

### Feedback Session Procedure

Participants attended the care and treatment clinics every 2 months to receive standard HIV care and ART medication refills, as per Tanzanian HIV treatment guidelines [6]. During the visit, participants in the 2 intervention arms (SMS and RTMM) received tailored feedback from the nurse counselor about their medication adherence according to SMS or RTMM. First, nurses asked participants whether they skipped any doses of ART and to what extent they had been able to take all doses of their ART in the past period. Next, the nurse provided participants with a graph (generated from SMS or RTMM) showing their level of adherence in percentages. Participants were asked to comment on their adherence behavior displayed on the graphs. Participants’ responses to the questions, their answers, comments, and any explanations for potential discrepancies between reported adherence and adherence based on SMS text messaging or RTMM were registered. The feedback was obtained using semistructured questionnaires and recorded on case report forms.

Viral load was measured at enrollment in all study participants. The lower detection limit of the viral load test was 20 copies/ml. According to Tanzanian HIV treatment guidelines, viral loads above 1000 copies/ml should be repeated after 3 months of intensified counseling on adherence to treatment and those below 1000 copies/ml should be repeated after a year. For participants defined as “cases,” we retrieved the viral load from their medical records at the beginning of the trial and at the end of the follow-up period of the trial.

### Case Selection and Interview

In this paper, we define cases as trial participants for whom there was a major discrepancy between self-reported adherence at the bimonthly study visit with the nurse and the adherence as indicated by SMS or RTMM (ie, patients reporting good adherence, 95% or higher, to the nurse, while SMS or RTMM indicated poor adherence, 75% or lower). All participants defined as cases were invited to an in-depth interview to explore whether they had encountered any technical or practical problems using SMS or RTMM. The interviews were conducted by the study investigator provided with a topic guideline, including perception of being monitored, technical issues, and privacy concerns (ie, concerns that others would see the RTMM device or read the content of the SMS text message, which could disclose the participant’s HIV status). The topic list was prepared by the study team.

### Analysis

For each participant defined as a case, we calculated the average level of adherence reported to the nurse at bimonthly study visits and the participant’s level of adherence according to SMS text messaging or RTMM over the 5 visits during the follow-up period. For self-reported adherence, we directly asked how many doses were missed, and from there we could calculate the participant’s adherence level. For adherence according to SMS text messaging or RTMM, we calculated the number of correct intakes (measured by correct openings of the pillbox or by the number of YES replies to the text message) divided by the number of prescribed intakes. We report the participants’ comments on discrepancies between self-reported and SMS/RTMM-based adherence, the explanations participants gave for the discrepancies, and possible technical problems, privacy concerns, or other issues that emerged during the interviews.

## Results

### Case Study Participants

By November 2018, 26 participants of 249 who started with the clinical trial had completed all 5 study clinic visits. Of the 26 participants, 12 were enrolled from Majengo sites and 14 from the Kilimanjaro Christian Medical Centre. As recorded by SMS text messaging or RTMM, adherence rates were higher than 75% in 20 participants. Adherence rates were lower than 75% in 6, but these participants self-reported adherence rates of 95% or higher and were thus considered to have major discrepancies between self-reported and SMS/RTMM-based adherence at each of the 5 study visits. For all cases, a median of 46% adherence based on SMS or RTMM was recorded. Three of these participants (all female) were enrolled in the SMS arm, and 3 (1 female and 2 male) were enrolled in the RTMM arm. All participants were on a twice-daily ART regimen. Two participants in the RTMM arm and 1 in the SMS arm had suppressed viral loads (Multimedia Appendix 1).

#### Case 1

The first case was a female living in urban Moshi who responded to 73 (43%) of 168 SMS text messages with the question of whether she took her medication. During study visits 1 to 5, the participant confirmed that she received SMS text message reminders on time, but she often did not respond to them; she said, “I think I lacked commitment in responding to SMS.”

She felt depressed because she was separated from her family. She had not expected that the nurses would find out that she was not responding to the SMS text messages. After she was confronted by the nurses about her low response rate to SMS text messages, she tried to reply to them more often. She mentioned that she had no difficulties taking her pills, despite not replying to the SMS text messages. To improve adherence to ART, she said,

I will set my phone alarm as extra reminder and involve the caregiver as well.

She did not have concerns about privacy and answered,

I have a smartphone and it’s easy to disable SMS notification as well as put a password. So, no one can see or have access to my SMS.

Therefore, this participant reported high levels of adherence to ART despite a low response rate to SMS text messages, and the low response rate appeared to be related to feelings of depression.

#### Case 2

Case 2 was an urban Moshi woman. Throughout visits 1 to 5, 186 SMS text messages were sent and delivered with the question about whether she took her medication. However, only 95 (51%) SMS text messages were responded to with, “Yes, I took it” and 91 (49%) SMS text messages were not responded to.

When confronted by the nurses about her low response rate to text messages during the feedback sessions, she mentioned that the main challenge that hindered her in replying to SMS text messages were her friends, who normally spent a lot of time with her, especially in the evening. She explained,

My friends are always in my room, especially in the evening; therefore, I am not feeling comfortable to respond to SMS.

When asked whether she had disclosed her HIV status to them, she said,

I’m scared to tell them, and with my age I still need them around. Also, none of my friends will believe I acquired HIV when I was born.

Of interest, she said, “Despite not responding to the SMS, I still take my medication.”

This participant also reported high levels of adherence to ART despite having a low response rate to SMS text messages. The main reason for the low response rate to SMS text messages was related to the fear of unwanted disclosure of her HIV status.

#### Case 3

In 2012, a 29-year-old woman living in urban Moshi started ART. During visits 1 to 5, 186 SMS text messages were sent and received, asking whether she took her medication. She only replied to 53 (28%) text messages and did not respond to 133 (72%).

During feedback sessions with the nurses, she insisted, “I never missed the medication intakes and I replied to the SMS.” She also said,

I am surprised to see that I did not reply to all SMS. I am wondering whether you are missing something as I believe that I managed to take about 80% of my pills.

However, in the interview with the study coordinator, she confessed,

I was not honest during the feedback sessions with the nurses. The reason for that is that I was depressed because my sister found out I was receiving SMS to remind me to take medication for HIV and then it became even worse when she started telling my neighbors that I will die soon.

When we asked if she thought the content of the SMS text messages should be changed, she said,

I don’t have a problem with the content. Nowadays I delete the SMS once I have replied to it so that no one will read them in case they gain access to my phone.

For this participant, the main reason for not replying to SMS was related to unwanted disclosure of HIV status and stigma surrounding HIV.

#### Case 4

Case 4 was a 58-year-old female living in rural Moshi who started ART in 2012 and was enrolled in the RTMM arm. According to RTMM, her level of adherence during the first 2 study months was 47%. After the nurses showed her this level of adherence during a feedback session, she said,

I never missed the medication intakes. I think the RTMM was not working properly. Therefore, I want to be provided with a new device.

The study nurse exchanged the old device with a new one. In the next feedback session, her level of adherence according to RTMM was still low, at 20%, despite her insistence that she never missed a medication intake.

During the interview with the study investigator, the participant mentioned that the device was easy to use, but charging it was a challenge because it did not have an alarm to indicate a low battery. At the beginning of the study, this participant lived in a rural area, where her adherence level was low, according to RTMM. After 10 months, she moved to an urban area and the device started to send daily signals, showing device openings indicative of a high level of adherence. The participant explained,

I have never missed my medication intakes; therefore, I was surprised why the device was not recording the openings. After I moved from my old house in the village, the device started to record the openings, so I thought the problem was network coverage.

The participant was happy to be monitored in real time, as it made her feel cared for. Privacy concerns were not an issue for this participant as she had disclosed her HIV status to her family.

For this participant, the main reason for the discrepancy between self-reported adherence and adherence generated by the device seemed to be related to adequate power, charging, and availability of network coverage.

#### Case 5

Case 4 was a 33-year-old male who lives in urban Moshi. He was enrolled in the RTMM arm and started ART in 2013. According to RTMM, his level of adherence to ART was 57%, on average, during the follow-up period of the study. This participant explained that his main problem using the RTMM device was concern about disclosure of his HIV status:

I have no problem with using the device except that I did not disclose my HIV status to my children and my co-workers. So sometimes it is difficult to open the device when I am with them. However, I always take my pills, which I kept outside the device.

This participant further explained,

I had difficulties carrying the device to my workplace and since I’m working late sometimes, I sometimes missed my evening dose of medication.

Thus, the main problems for this participant were related to unwanted disclosure of HIV status and difficulties incorporating the use of the device in daily activities.

#### Case 6

The sixth case was a 21-year-old man. With an average of 60% ART adherence during the follow-up of the study, he acknowledged missing his medication due to the lack of an alarm on the RTMM device. He explained,

When I’m home with the device, I normally forget to open it…Sometimes I wish the device should have an alarm to notify me to open it for my medication intake.

He also said,

Currently I depend on other reminders, such as news time hours and the alarm of the wall clock.

Before this participant enrolled in the study, he used to set an alarm on his mobile phone as reminder to take his medication. Now he says,

Since the device does not give an alarm, sometimes I missed my medication intakes as there is nothing to ring as alarm. However, as soon I receive the SMS reminder, I take my medication.

Therefore, for this participant, the main reason for poor adherence recorded by the device was that the normal strategy that he used to remind himself to take his medication was interrupted when entering the study, and he needed to adopt a new strategy. As the SMS text message reminder comes late, the participant might indeed be adherent but not on time.

## Discussion

### Principal Findings

This case series explored, in detail, 6 participants who had major discrepancies between their self-reported and digitally monitored adherence. This paper illustrates the challenges in using mHealth, which emerged during an intervention trial using SMS text message reminders and RTMM to improve adherence to ART among people living with HIV in the Kilimanjaro region in Tanzania. Interviews were conducted to determine whether the discrepancies were triggered by difficulties in using the interventions, poor network, power failures, potential stigma, or other reasons.

One remarkable observation was that it is important to distinguish between adherence to proper use of the digital adherence monitoring device and adherence to treatment. Patients enrolled in the SMS arm were expected to respond to each question they received by SMS text message. However, of all the SMS text messages that were delivered, on average, only 46% postintake text messages were responded to. The results showed that the postintake SMS text message was not triggering the trial participants to respond well to the messages. This finding seems to be in line with a previous study reporting that implementation of mHealth interventions in low-income countries can be complicated if end users have difficulties adapting to new technologies due to lack of experience or psychosocial barriers, which may lead to inadequate use of the technology (ie, limited responding to messages or incorrect use of the device for medication intake) [9].

Another remarkable finding was that several participants considered using additional ways to remind themselves to take their medication, such as using a phone alarm. We found this striking, as sending electronic reminders to participants was part of both intervention arms, yet participants needed additional tools to help them remember to take their medicine on time.

One participant insisted that she took her medication even when the RTMM device was replaced with a new one, but the new device continued to indicate poor adherence. This discrepancy turned out to be caused by the device not finding a network, and it was therefore unable to properly transmit the signal to indicate the medication intake event. As such, each time the device was opened, it was not recorded in the system. However, after the participant moved to an area with a network, the device was able to send the signal on a daily basis. This underlines that adequate network coverage is a prerequisite for the feasibility of mHealth.

In our previous study in the Kilimanjaro region [8], we also encountered the problem of network instability due to bad coverage that led to delays and failures in delivering reminder SMS text messages among participants residing in several remote villages. Despite the technical challenges that were addressed by interviewing participants, we believe that using mHealth and integrating it with the existing health system is possible, but it would require stability of several factors such as network coverage and power supply [9,10]. We therefore suggest that future studies on mHealth interventions in resource-limited settings may benefit from involving other stakeholders, especially mobile network providers, since they have a vital role in providing stable network coverage necessary for delivering such interventions.

Psychosocial barriers, including mental health issues and lack of social support, have been shown to result in poor use of interventions due to fear of HIV disclosure within the family or at workplaces. Findings from a previous study in Kenya showed that SMS text messaging had better effect on treatment adherence for those who received SMS text messages and who reported a high level of social support [11]. Therefore, it is important to take into account mental health issues when applying digital tools by, for example, providing support through messages or using the tool to increase communication about mental health issues during clinic visits.

Privacy was a main issue for participants who did not disclose their HIV status to others. This finding is consistent with that of studies in South Africa and a study in Kenya that showed that some participants had concerns that monitoring was intruding on their privacy [12,13]. The use of neutral messages that do not refer to topics related to sickness or medication could overcome the problem of unwanted disclosure. In addition, participants in the South African study had concerns that the size and design of the pillbox would reveal their HIV status to the community, especially when they needed to open it in the vicinity of others. However, our pilot study in Kilimanjaro [8] showed a different result, as most participants were happy with the color and size of the device.

Case 6 illustrates the importance of ensuring that an intervention intended to improve adherence does not interfere with a person’s strategy to enhance their level of adherence. Despite that we told our participants to keep using their normal ways of reminding themselves to take their medication, this participant decided to quit using his phone alarm. Although he declared that the reminder SMS text message served as an alarm to him, it did not lead to improved medication intake according to the device. We cannot say that using the device hampered medication intake, as this participant was judged to be nonadherent at enrollment. Unfortunately, it was not clear why this patient was not adherent. It is possible that the participant was still not willing to disclose to the interviewer that he was not taking his medication or not taking it on time.

Two participants had suppressed viral loads after being exposed to the intervention for several months, despite having low levels of adherence as indicated by responses to text messages or by recorded RTMM device openings. It remains unclear whether these participants were adherent to taking their medication but nonadherent to the proper use of the intervention (ie, responding to SMS text messages or opening the RTMM device). This difficulty in distinguishing between adherence to treatment and adherence to proper use of an intervention or device is not unique to the 2 mHealth interventions that were used in this study. Similar findings have been reported in 2 studies conducted in Kenya [11,12]. Another explanation for the suppressed viral loads might be that levels of adherence were incorrectly recorded due to technical problems. To be able to distinguish between true low adherence to treatment and low adherence to the intervention combined with technical problems, drug levels in the blood could give more insight. Because it was not possible to measure plasma concentrations of antiretroviral drugs in this study, we could not verify whether these participants were nonadherent to their medication intake or nonadherent to using their device.

### Conclusion

This case series explored the reasons underlying major discrepancies between self-reported adherence and digitally monitored adherence that occurred in 6 participants in our mHealth intervention trial. Based on our findings, we can make a number of recommendations. First, network coverage at the participant’s home must be ensured before implementation. Second, neutral messages (eg, “Hello, this is your friend from the REMIND Study. I hope you are doing well.”) should be used to avoid unwanted disclosure of HIV status. Third, participants should be advised to continue using their usual reminder cues. Fourth, a triage system could be developed to determine whether a patient is ready to use such mobile interventions to prevent the paradoxical situation that an intervention intended to improve treatment adherence actually worsens adherence. Fifth, researchers should attempt to distinguish between adherence to the use of a monitoring device and adherence to treatment. Last, our findings serve as a reminder of the paramount importance of psychosocial support in the context of providing HIV treatment and care.

## Appendix

Multimedia Appendix 1 Summary of case characteristics.

## Abbreviations

ART: antiretroviral therapy
mHealth: mobile health
RTMM: real-time medication monitoring
