# Supplementary material for: Technical and Psychosocial Challenges of mHealth Usage for Antiretroviral Therapy Adherence Among People Living With HIV in a Resource-Limited Setting: Case Series
Source: JMIR Form Res. 2020 Jun 10;4(6):e14649. doi: 10.2196/14649 (PMC7315367; doi:10.2196/14649)
Supplement: Multimedia Appendix 1 [file formative_v4i6e14649_app1.docx]

| Case | mHealth intervention | Age, gender | ART regimen | Self-reported adherence | Adherence according to SMS/RTMM | Viral load (copies/mL) | | |
| --- | --- | --- | --- | --- | --- | --- | --- | --- |
|  |  |  |  |  |  | Enrollment | Month 3 to 6 | Month 12 |
| 1 | SMS | 20-year-old female | On 2^nd^ line regimen  Started ART in 2011 | 95 | 43% | 1927 | 2627 | 129 |
| 2 | SMS | 19-year-old female | On 1^st^ line regimen  Started ART in 2008 | 97 | 51% | 47587 | Not assessed due to missed clinic visit | 6679 |
| 3 | SMS | 29-year-old female | On 1^st^ line regimen  Started ART in 2012 | 100 | 28% | 367 | No test done as VL was <1000 | Not detectable |
| 4 | RTMM | 58-year-old female | On 1^st^ line regimen  Started ART in 2012 | 100 | 33% | 16106 | <20 | Not detectable |
| 5 | RTMM | 33-year-old male | On 1^st^ line regimen  Started ART in 2013 | 97 | 57% | 2863 | 67 | 12447 |
| 6 | RTMM | 21-year-old male | On 1^st^ line regimen  Started ART in 2005 | 100 | 60% | 739 | <20 | Not detectable |

Appendix 1. Summary of Case characteristics
